# Garlic arrests MDA-MB-435 cancer cells in mitosis, phosphorylates the proapoptotic BH3-only protein Bim_EL_ and induces apoptosis

**DOI:** 10.1038/sj.bjc.6602537

**Published:** 2005-04-12

**Authors:** T Lund, T Stokke, Ø E Olsen, Ø Fodstad

**Affiliations:** 1 Department of Tumor Biology, Institute for Cancer Research, The Norwegian Radium Hospital, Montebello, Oslo 0310, Norway; 2 Department of Radiation Biology, Institute for Cancer Research, The Norwegian Radium Hospital, Montebello, Oslo 0310, Norway

**Keywords:** garlic, mitosis, apoptosis, Bim_EL_

## Abstract

Components of garlic (*Allium sativum*) can cause disruption of microtubules, cell cycle arrest, and apoptosis in cancer cells. We show here that a water-soluble extract of garlic arrested MDA-MB-435 cancer cells in mitosis and caused apoptosis. The proapoptotic BH3-only, bcl-2 family protein Bim_EL_, which in healthy cells can be tightly sequestered to the microtubule-associated dynein motor complex, was modified after garlic treatment. The main effect of garlic on Bim_EL_ was a considerable increase in a phosphorylated form of the protein. This phosphorylation(s), probably partly dependent on c-jun N-terminal kinase activity, promoted mitochondrial localisation of Bim_EL_. Furthermore, inhibition of extracellular signal-regulated kinases 1/2 increased the amount of another form of Bim_EL_ present in the mitochondrial cellular fraction. Treatment of cells with the garlic compound diallyl disulphide had similar effects on Bim_EL_. The results indicate that the apoptotic effect of garlic and a combination of garlic and the inhibitor of extracellular signal-regulated kinases 1/2 in MDA-MB-435 cells partly is due to modifications that are necessary for translocation of the proapoptotic protein Bim_EL_ to mitochondria where it executes its proapoptotic function.

Different compounds of garlic have been shown to have antiproliferative effects on breast, colon and leukaemic cancer cells (Agarwal, 1996; Dirsch *et al*, 1998; Knowles and Milner, 1998, 2000; Nakagawa *et al*, 2001; Shirin *et al*, 2001). These effects were due to cell cycle arrest in G_2_ and mitosis and apoptosis. It is shown that ajoene, an organosulphur compound originating from garlic, activated signal pathways that may have anti- and proapoptotic effects, respectively (Antlsperger *et al*, 2003). Importantly inhibition of extracellular signal-regulated kinases (ERK) 1/2 amplified the ajoene-induced cell death and this combination could therefore be interesting in cancer treatment. Furthermore, diallyl disulphide (DADS) and diallyl trisulphide (DATS), oil-soluble garlic compounds induce apoptosis that is dependent on the redox-sensitive c-jun N-terminal kinase (JNK) pathway (Filomeni *et al*, 2003; Xiao *et al*, 2004). Recently, it was discovered that another compound derived from garlic, S-allylmercaptocysteine (SAMC) causes microtubule depolymerisation by interacting directly with tubulin (Xiao *et al*, 2003). This effect is similar to the effect of microtubule interacting drugs that are already used in cancer therapy and encourage further application of garlic and garlic compounds in cancer treatment. Molecular events related to apoptosis downstream of activation of ERK and JNK and microtubule disturbance are scarcely characterised, but it has been suggested that phosphorylation of bcl-2 by JNK could have a proapoptotic effect (Xiao *et al*, 2004).

The proapoptotic protein Bim (O’Connor *et al*, 1998), which belongs to the BH3-only group of the bcl-2 family (Huang and Strasser, 2000; Cory and Adams, 2002), is a possible candidate for transferring apoptotic signals resulting from microtubule damage. Apoptosis after treatment of cells with the microtubule stabilising agent taxol has, indeed, been shown to involve Bim (Puthalakath *et al*, 1999). An isoform of Bim, Bim_L_, is sequestered away from its apoptotic target, the mitochondria, in healthy cells by interaction with dynein light chain (DLC-1/LC8) of the microtubule-associated dynein motor complex. Another isoform, Bim_EL_, is reported to interact with *α*-tubulin in addition to DLC-1/LC8 (Chen and Zhou, 2004), suggesting that Bim_EL_ is sequestered firmer than Bim_L_ to microtubules. Some apoptotic agents, among them taxol, causes translocation of Bim_L_ from the dynein motor complex to the mitochondria. When translocated, Bim is suggested to either interact with and inactivate antiapoptotic bcl-2 proteins or directly interact with and activate the proapoptotic bcl-2 proteins bak and bax (Marani *et al*, 2002). Downstream effects of proapoptotic bcl-2 proteins are release of cyt *c* in cytosol and activation of caspase-9.

Phosphorylations are probably very important for regulation of the apoptotic activity of Bim_L_ and Bim_EL_. Analysis of human Bim_EL_ using the Scansite program (http://scansite.mit.edu/) predicts 11 possible phosphorylation sites with medium stringency, and many of these sites are phosphorylated *in vitro* and probably *in vivo* (Biswas and Greene, 2002; Lei and Davis, 2003; Ley *et al*, 2003; Luciano *et al*, 2003; Putcha *et al*, 2003; Marani *et al*, 2004). It has been suggested that phosphorylation caused by JNK, in the domain that interacts with LC8 (Thr-56 in Bim_L_ corresponding to Thr-116 in Bim_EL_), is necessary for its apoptotic function (Lei and Davis, 2003). Furthermore, phosphorylation of Ser-69 of human Bim_EL_, which is specific for this isoform, by ERK1/2 is reported to have an antiapoptotic function by promoting degradation of Bim (Luciano *et al*, 2003). In contrast, phosphorylation of the same site by JNK is reported to be necessary for the apoptotic function of Bim_EL_ in neurons (Putcha *et al*, 2003).

In the present study, we have used a human metastatic cancer cell line, MDA-MB-435, to identify molecular effects of garlic that may be important for apoptosis. Garlic treatment of these cells gave a strong mitotic arrest and apoptosis, and both cell survival and cell death-signaling pathways were activated. Post-translatory modifications of the BH3-only bcl-2 family protein Bim, that seem to be important for apoptosis, were characterised.

## MATERIALS AND METHODS

### Cell culture

The human cancer cell line MDA-MB-435, which carries a mutated p53 gene (266 GGA-GAA) (O’Connor *et al*, 1997), was used. This cell line has widely been accepted as originating from a breast cancer, but some recent publications have suggested that the cells originate from a melanoma (Ellison *et al*, 2002). These cells and MCF-7 breast cancer cells were grown in monolayer cultures in RPMI 1640 medium (Gibco, Grand Island, NY) supplemented with 10% fetal calf serum, 20 mM HEPES buffer pH 7.5 and 2 mM L-glutamine (Gibco).

### Preparation of garlic extracts, quantification of thiosulphinates and use of DADS

Extracts were prepared from garlic bulbs (*Allium sativum* var. *sativum* (Oswega white)) (a gift from Dr Gowsala P Sivam) essentially as described by Sivam *et al* (1997). Garlic cloves were crushed with a morter and diluted in water 1 : 2. Undissolved material was removed by centrifugation at 15 000 × **g** for 20 min, and the supernatant was filtered through a 0.2 *μ*m filter. The concentration of thiosulphinates in the extracts was measured by the method of Han *et al* (1995). This method utilises that two molecules of allicin (the main component of crushed garlic) reacts with two molecules of cysteine to form two molecules of SAMC. The reduction of cysteine is determined by measuring the amount of 2-nitro-5-thiobenzoate formed after reaction with 5,5′dithio-bis-(2-nitrobenzoic acid). Diallyl disulphide (Fluka) at a concentration of 5.5 M was dissolved in DMSO and used at a final concentration of 50 *μ*M in cell cultures (Filomeni *et al*, 2003).

### MTS assay

Viable cells were measured by a colorimetric assay composed of solutions of a tetrazolium compound (3-(4,5-dimethylthiazol-2-yl)-5-3(3-carboxymethoxyphenyl)-2-(4-sulphophenyl)2H-tetrazolium, inner salt; MTS) (Promega, Madison, WI) and an electron coupling reagent (phenazine metthosulphate; PMS). MTS is bioreacted by cells into a formazan product that is soluble and the absorbance of the formazan at 490 nm is measured directly. Cells were seeded at a density of 4000 cells/well in a 96-well plate. After 24 h, the cells were added into fresh medium containing different concentrations of garlic extract and allowed to grow for additional 24 h before adding the MTS reagents. Triplicate measurements with different garlic concentrations were performed, and the concentration that gave a 50% reduction in the number of living cells (IC_50_) was estimated.

### TUNEL-staining for flow cytometry

Terminal deoxynucleotidyl transferase (TdT)-mediated dUTP-biotin nick end labelling (TUNEL) method (Gavrielli *et al*, 1992), which detects 3′-OH ends of newly cleaved DNA, was used to quantify DNA fragmentation by flowcytometry. Both adherent and floating cells were collected and fixed in 100% methanol for at least 1 h at −20°C. The cells were rinsed in PBS and incubated for 45 min at 37°C in 50 *μ*l of a solution containing 5 units TdT, 0.5 nM biotin-16-dUTP (Boehringer Mannheim, Mannheim, Germany), 0.1mM dithiothreitol, 1.5 mM CoCl_2_ and 5 *μ*l 10 × reactionbuffer (Boehringer Mannheim). After this incubation, the cells were washed in PBS, then in PBS containing 0.1% Triton X-100 and incubated for 30 min at 0°C in 50 *μ*l PBS/Triton X-100 containing 1 : 50 streptavidin-FITC (Amersham, Aylesbury, UK). Thereafter, the cells were washed in PBS/Triton X-100 and stained with Hoechst 33258 (2 *μ*g ml^−1^) in PBS/Triton X-100 (Stokke *et al*, 1998).

### Cell cycle distribution

All steps were performed at 0°C. Harvested cells were washed once in PBS and resuspended in 750 *μ*l 0.1% Nonidet P40, 150 mM NaCl, 0.5 mM EDTA, 0.1 mM phenylmethylsulphonyl fluoride, 10 mM Na-phosphate (pH 7.4). After 5 min, cells were vortexed and 250 *μ*l 4% paraformaldehyde was added. Cells were fixed for at least 1 h, then spun down and stained with 12.5 *μ*g ml^−1^ propidium iodide (Calbiochem, La Jolla, CA) in the presence of 100 *μ*g ml^−1^ RNase A (Pharmacia, Uppsala, Sweden) in 0.1% Nonidet P40, 150 mM NaCl, 0.5 mM EDTA, 0.1 mM phenylmethylsulphonyl fluoride, 10 mM Na-phosphate (pH 7.4). Propidium iodide stained cells were measured in a FACScan flow cytometer (Becton Dickinson). The simutaneous measurement of fluorecence and light scattering allows discrimination of G_1_, S, G_2_ and M phase cells since mitotic cells bind more dye and scatter less light than G_2_ cells (Larsen *et al*, 1986). The percentage of mitotic cells was calculated by employing a region as shown in Figure 2 (labelled M). The distribution of interphase cells between the G_1_, S, and G_2_ phases of the cell cycle was determined from the PI fluorescence histograms (Modfit, Verity Software House, Topsham, ME) after exclusion of the mitotic cells. The percentages of interphase cells were renormalised to account for the presence of mitotic cells by multiplying with the factor: 1−(% mitotic cells/100).

### Immunoprecipitations and *in vitro* kinase reactions

For cyclin-dependent kinase Cdc2 (Cdk1) reactions, the cells were lysed in a lysis buffer containing 20 mM Tris, pH 8.0, 1 mM EDTA, 400 mM NaCl, 0.5% Nonidet P-40, 5 mM NaF, 1 mM phenylmethylsulphonyl fluoride, 50 kallikrein-inactivating units of aprotinin, 10 *μ*g ml^−1^ leupeptin, 10 *μ*g ml^−1^ trypsin inhibitor, 0.5 mM Na-orthovanadate, 20 mM *β*-glycerophosphate. In all, 500 *μ*g cell lysate was incubated with 2 *μ*g of anti-cyclin B1 at 4°C overnight to precipitate Cdc2/cyclin B1 complexes. Immunocomplexes were captured by rocking the reaction mixture with 25 *μ*l Protein G Sepharose 4B fast flow bead slurry (Sigma, St Louis, MD) at 4°C for 2 h. Protein G Sepharose beads were extensively washed, and used in kinase reactions in the presence of 10 *μ*g histone H1 (Upstate, Lake Placid, NY), 50 *μ*M ATP, and 2.5 *μ*Ci of (*γ*-^32^P)ATP in a kinase buffer consisting of 50 mM HEPES, pH 7.5, 5 mM MgCl_2_, 2.5 mM MnCl_2_, and 1 mM dithiothreitol.

### Cellular fractionation

Cytosolic and mitochondrial fractions were prepared using the mitochondrial/cytosol fractionation kit (Alexis Biochemicals, San Diego, CA) according to the manufacturer's instructions. In short, cells were harvested, washed with PBS and resuspended in a cytosol extraction buffer and incubated for 10 min. Next, cells (500 *μ*l) were homogenised by 10 passages trough a 26-gauge needle. Lysis of the cells were examined and confirmed by microscopy. Nuclei and debris were pelleted by centrifugation at 700 **g** for 10 min. The supernatant was then centrifuged at 10 000 **g** for 30 min. The new supernatant (500 *μ*l) was collected as the cytosolic fraction. The pellet was resuspended in 100 *μ*l mitochondrial extraction buffer and saved as the mitochondrial fraction. COX IV and *α*-tubulin were used in Western blot as markers for the fractions containing mitochondria and cytosol, respectively.

### Inhibition of activities of ERK1/2 and JNKs

Approximately 1 × 10^6^ cells/T25 flasks were grown for 1 day before adding garlic or inhibitors. MEK1 inhibitor PD 98059 (2′-amino-3′methoxyflavone; Sigma), used to inhibit the activation of ERK1/2, was added (12.5 *μ*M final concentration) 1 h before addition of garlic. JNK Inhibitor II, SP600125 (Calbiochem) (final concentration 20 *μ*M) was added 3 h before harvesting the cells.

### Western blot analysis

Cells in T25 flasks were harvested before confluence and lysed in a lysis buffer containing 50 mM Tris, pH 7.5, 150 mM NaCl, 0.1% Nonidet P-40, 5 mM NaF, 1 mM phenylmethylsulphonyl fluoride, 50 kallikrein-inactivating units of aprotinin, 10 *μ*g ml^−1^ leupeptin, 10 *μ*g ml^−1^ trypsin inhibitor, 0.5 mM Na-orthovanadate, 20 mM *β*-glycerophosphate, and supernatant proteins after centrifugation of the lysates were analysed by SDS–PAGE. Protein concentration was measured by the Bradford method (Bradford, 1976). The same amount of protein (80 *μ*g) was applied to each well on a gel unless else is stated. Separated proteins were transferred to a polyvinylidene difluoride membrane (Millipore Corporation, MA, USA). The antibodies used in this study were: rabbit anti-phospho (Thr202/Tyr204) p44/42 MAP kinase (ERK1/2), anti p44/42 MAP kinase (ERK1/2), anti-phospho (Thr183/Tyr185) JNK and anti JNK (all Cell Signaling, Beverly, MA), rabbit anti-Bim (Chemicon, Victoria, Australia), rabbit anti-p85PARP (Promega), rabbit anti phospho-c-jun (Calbiochem), mouse anti-cyclin B (Upstate), mouse anti-cytochrome (cyt) *c* oxidase subunit IV (COX IV) (Molecular Probes, Eugene, OR), mouse anti-caspase-9 (R&D systems, Minneapolis, MN), mouse anti-cyt *c* (Santa Cruz, CA), and mouse monoclonal anti *α*-tubulin (Oncogene research products). Nonspecific binding sites were blocked according to the recommendations of the manufacturer of each antibody and the blots were incubated with primary antibodies over night at 4°C. After washing in solutions recommended by the manufacturer the blots were incubated for 1 h with either HRP-conjugated goat anti-rabbit or rabbit anti-mouse antibodies (Daco, Carpintera, CA) diluted 1 : 5000. Detection of protein bands was developed with a chemiluminescent substrate (ECL Western blotting detection reagents, Amersham) according to the description of the manufacturer. The results shown are representative for at least two different experiments. Quantitative estimates were obtained from scanning in a densitometer (Molecular Dynamics).

## RESULTS

**Figure 1:**
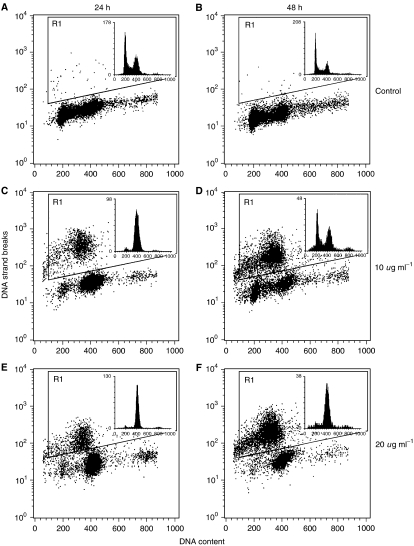
The histograms for DNA fragmentation show biotin-dUTP content as a measure of DNA strand breaks (ordinate) *vs* DNA content (abscissa) and cells undergoing apoptosis are enclosed in window R1. The histograms for DNA distribution (upper right corner) show DNA content (abscissa) against number of cells (ordinate). The data are from untreated cells after 24 h (**A**) and 48 h (**B**), cells treated with 10 *μ*g ml^−1^ thiosulphonates for 24 h (**C**) and 48 h (**D**) and cells treated with 20 *μ*g ml^−1^ thiosulphonates for 24 h (**E**) and 48 h (**F**).

Measurements of the effect of the garlic extract on the growth of MDA-MB-435 cells by an MTS-assay showed a reduction of the number of viable cells with an IC_50_ concentration of approximately 15 *μ*g ml^−1^ thiosulphinates after 24 h treatment with garlic extract. Thiosulphinate concentrations slightly below and above this concentration were used for measurements of cell cycle distribution and apoptosis. TUNEL staining and flow cytometry, giving a measure of DNA fragmentation, were used to quantify apoptotic cells. This method also gives an estimate of the number of cells undergoing apoptosis in relation to their position in the cell cycle. After 24 h treatment with garlic extracts containing 10 and 20 *μ*g ml^−1^ (Figure 1C and E) of thiosulphinates, 17.9% (s.e.m.±2.9) and 16.2% (s.e.m.±2.7) of the cells, respectively, were scored as apoptotic (average of triplicate experiments), and the corresponding values after 48 h were 46.6% (s.e.m.±10.2) and 56.2% (s.e.m.±13.3) (Figure 1D and F). With 5 *μ*g ml^−1^ thiosulphinates only 1.2–2.7% was apoptotic, while 40 *μ*g ml^−1^ resulted in mostly dead cells and cell debris. The cells were arrested in G_2_/M phase (see inserts in Figure 1). Cells with unfragmented DNA (nonapoptotic) were chosen for the analysis of cell cycle distribution due to the possibility of leakage of small DNA fragments from apoptotic cells. It is difficult to determine exactly the cell cycle position of the apoptotic cells, but it seemed to be that not only cells arrested G2/M phase were undergoing apoptosis.

**Figure 2:**
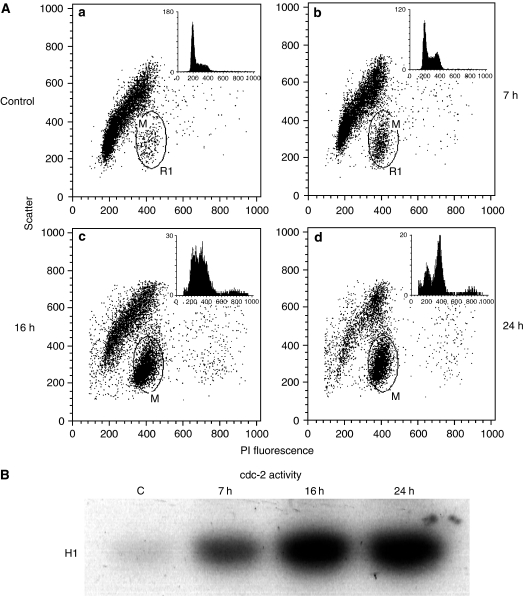
Cell cycle distribution of cells treated with garlic and activation of cdc-2 kinase. (**A**) Control cells (a), cells treated with 20 *μ*g ml^−1^ garlic extract for 7 h (b), 16 h (c) and 24 h (d). The positions for cells suggested to be mitotic are marked by circles and the percent of gated cells were calculated. The histograms for DNA distribution of nonmitotic cells (upper right corner) show DNA content (abscissa) against number of cells (ordinate). (**B**) Activity of Cdc2-kinase using histone H1 as substrate.

Microscopy of cells stained with Hoechst showed that a high fraction of cells, treated for 24 h with garlic, had visible chromosomes indicating the presence of mitotic cells (not shown). In order to distinguish between cells in G_2_ and mitosis and to measure the percent of cells in each phase nuclear suspensions of MDA-MB-435 cells were fixed with paraformaldehyde and stained with propidium iodide in the presence of RNase. This method allows discrimination of G_2_ and M phase cells due to differences in dye-binding and light scattering (Larsen *et al*, 1986). Flow cytometry showed accumulation of cells with a forward scatter and PI-fluorescence typical for mitotic cells after treatment with the garlic extract (Figure 2). Cells treated with garlic extract (20 *μ*g ml^−1^ thiosulphonates) for 7 h were arrested both in G_2_ and in M phase. The cells treated for 16 and 24 h had escaped the G_2_ arrest and 52.4% (s.e.m.±1.8) and 62.0% (s.e.m.±3.2), respectively, of the cells were mitotic, reflecting a strong mitotic arrest.

Cdc2 kinase is normally activated at the G_2_–M transition and the activity is high during most of the mitotic phase. It was therefore of interest to determine the activity of this kinase after garlic treatment. Cdc2/cyclinB1 complexes from cell lysates were immunoprecipitated with anti-cyclinB antibodies and the kinase activity was recorded with histone H1 as substrate. The kinase activity of the cells treated with garlic for 7, 16 and 24 h was approximately 4, 11 and 12 times higher than those of the control cells, respectively (Figure 2B), further indicating a mitotic arrest.

**Figure 3:**
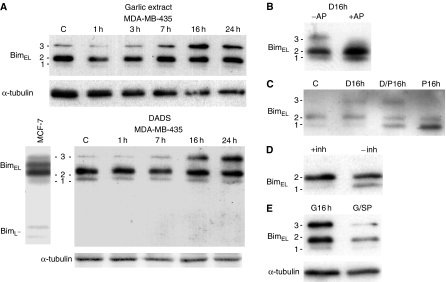
Bim from lysates of MDA-MB-435 cells. (**A**) Western blots, using antibodies against Bim, of lysates from MDA-MB-435 cells isolated at different times after addition of garlic or DADS and lysates from untreated MCF-7 cells. (**B**) Western blot of lysates treated with (+AP) and without (−AP) alkaline phosphatase for 30 min. The cells were previously treated for 16 h with garlic. (**C**) Western blot of lysates from control cells (C) and cells treated with DADS (D16h), a combination of DADS and PD98059 (D/P16h) and PD98059 (P16h) for 16 h. (**D**). Western-blot of lysates from cells lysed with (+inh) and without (−inh) phosphatase inhibitors in the lysis buffer. (**E**) Western blot of lysates from cells treated with (G/SP) and without (G16h) JNK inhibitor II, SP60012, for 3 h. The cells were previously treated for 13 h with garlic.

The proapoptotic BH3-only, bcl-2 protein, Bim, has previously been shown to be important for apoptosis induced by the microtubule interfering agent, taxol, which also arrests cells in mitosis (Puthalakath *et al*, 1999). Different phosphorylations by ERK1/2 and JNK probably regulate the function of Bim and translocation of the molecule from the dynein complex in cytosol to mitochondria is necessary for its apoptotic function in some cell lines. We investigated if the garlic extract had an effect on Bim. Bim from MDA-MB-435 cells were compared with Bim from MCF-7 cells (Figure 3A), which express the isoforms Bim_L_ and Bim_EL_ (O’Reilly *et al*, 2000). Bim_EL_ is reported to migrate as multiple bands of 24–26 kDa when examined by immunoblot analysis (Putcha *et al*, 2003; Wang *et al*, 2004), probably due to different degree of phosphorylation. Western blot analyses of lysates of MDA-MB-435 cells, using a polyclonal antibody recognizing an N-terminal epitope present in Bim_S_, Bim_L_ and Bim_EL_, gave three bands at the positions of Bim_EL_ of MCF-7 cells (approximately 25 kD) as expected for Bim_EL_. Band 3 appeared in increased levels when cells were treated with garlic and the garlic component DADS, respectively, for 16 and 24 h (Figure 3A). After treatment of lysates with alkaline phosphatase (Figure 3B, lane +AP), this band disappeared, indicating that it contained a phosphorylated form of Bim_EL_. We also investigated if the MEK/ERK-kinase inhibitor PD98059, alone or in combination with garlic extract or DADS had effect on the band pattern of Bim and found that band 1 was increased and band 2 was reduced as a result of the inhibitor (Figure 3C). The level of band 3 was, however, not reduced after addition of PD98059 (Figure 3C, D/P16h), indicating that another kinase than ERK was responsible for the phosphorylation in this form of Bim_EL_. Omission of phosphatase inhibitors (−inh) in the lysis buffer also resulted in the decrease of band 2 and increase of band 1 (Figure 3D). Together, this indicates that band 2 also was a phosphorylated form of Bim_EL_ and that the phosphorylation at least in part was dependent on ERK1/2 activity. Bim_EL_ in band 2 was, however, relatively unaffected by alkaline phosphatase. This could be because the conformation of the protein makes some of the phosphorylation sites inaccessible for that phosphatase or that some other proteins are associated with Bim_EL_ in agreement with observations by Seward *et al* (2003) on the same protein. Cells were also treated with an inhibitor of JNK, SP600125. This inhibitor partly inhibited garlic-induced phosphorylation of Bim_EL_ in band 3 (G/SP) and decreased the total level of the protein (Figure 3E).

**Figure 4:**
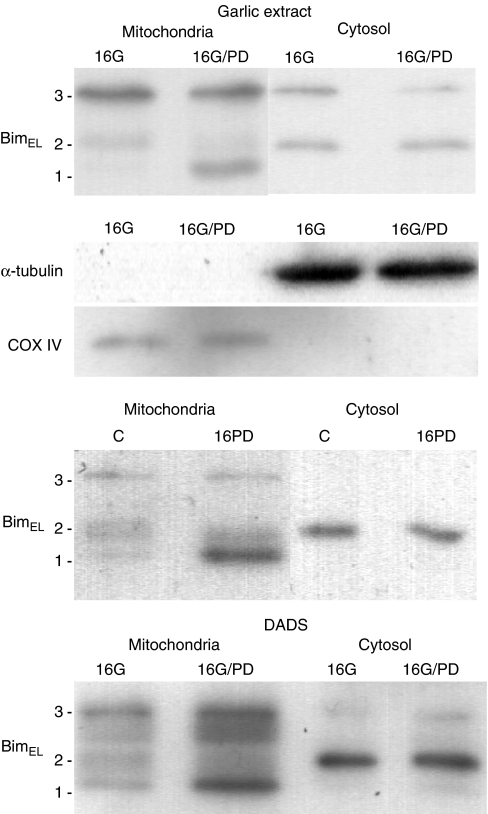
BimEL present in mitochondrial and cytosolic cellular fractions. Untreated control cells (C) and cells treated with garlic (16G), DADS (16D), a combination of garlic and PD 98059 (16G/PD, a combination of DADS and PD98059 (16D/PD) and only PD98059 (16PD) for 16 h were fractionated in mitochondrial and cytosol fractions. Western blots of the different fractions are shown. Antibodies against *α*-tubulin and COX IV were used to verify the fractionations.

In order to determine if the modifications of Bim_EL_ were of importance for the distribution of this protein between cytosol and mitochondria, cytosolic and mitochondrial cellular fractions were analysed by Western blotting (Figure 4). The presence of COX IV only in the mitochondrial fraction and *α*-tubulin only in the cytosolic fraction proved the efficiency of the fractionation. The three different forms (band 1, 2 and 3) of Bim_EL_ were distributed differently between the different fractions. It was seen that the ratio between mitochondrial and cytosolic Bim_EL_ was highest for the form in band 1 and lowest for the form in band 2. This means that both the specific phosphorylation caused by the garlic extract and DADS (enhancing band 3) and the dephosphorylation caused by PD98059 (enhancing band 1) will enhance the level of Bim_EL_ in the mitochondrial fraction.

**Figure 5:**
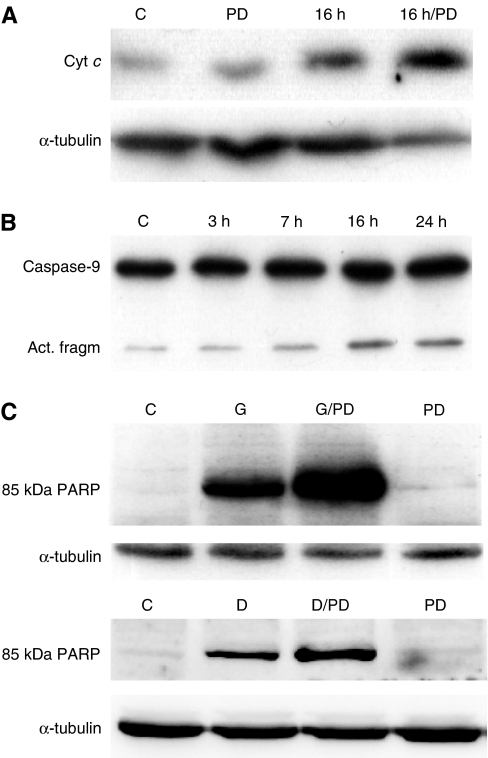
Changes in apoptotic markers downstream of Bim. (**A**) The amount of cyt *c* released into the cytosol after treating cells with garlic extract and a combination of garlic and PD98059 for 16 h (Western blot). (**B**) Activation of caspase-9 after treating cells with garlic extract for different times (Western blot). The lower caspase-9 band represents an activated form. (**C**) Quantification, by Western blotting, of the amount of a caspase-specific cleavage product of PARP (85 kDa PARP) after treating cells with garlic (G) or DADS (D) and combinations of garlic and PD98059 (G/PD) and DADS and PD98059 (D/PD) for 24 h.

Possible downstream effects of proapoptotic bcl-2 proteins are release of cyt *c* from mitochondria to cytosol and activation of caspase-9. If the observed modification of Bim_EL_ and translocation of Bim_EL_ to mitochondria really were apoptotic events, an increase in cytosolic cyt *c* followed by activation of caspase-9 could therefore be expected. A slight increase in cyt *c* in the cytosolic fraction after addition of garlic and PD98059 and garlic alone was indeed found and the increase was highest after the addition of the combination (Figure 5A). An increase in an activated form of caspase-9 (Figure 5B) was detected at the time when band 3 of Bim_EL_ increased.

To characterise further downstream apoptotic effects of the garlic extract, the level of a caspase-specific cleaved form of PARP, which is regarded as a hallmark of apoptosis, was measured. This marker increased when the cells were treated for 24 h with the garlic extract and was still higher when the combination of garlic and the MEK/ERK-inhibitor PD98059 was used (Figure 5C). PD 98059 alone gave no enhancement in cleaved PARP. A similar effect was also observed when cells were treated with DADS (Figure 5C).

**Figure 6:**
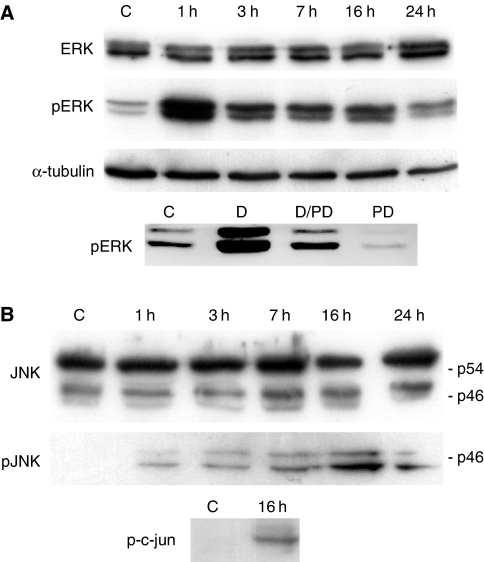
Activation ERK and JNK during treatment of MDA-MB-435 cells with garlic. (**A**) Western blot showing the amount of activated ERK1/2 (pERK) (antibodies against phospho-ERK1/2 (Thr202/Tyr204) were used) and total ERK (labelled ERK) at different times after addition of garlic. The amount of pERK after addition of PD98059 and DADS were also measured. (**B**) Western blot showing the amount of activated JNK (pJNK) and total JNK (labelled JNK) at different times after addition of garlic. Two main forms of JNK (p54 and p46) and a minor form below p46 were detected. The phospho-specific antibody recognised p46 and the form below.

We also investigated if ERK and JNK were activated during treatment of cells with garlic, since these kinases seemed to be involved in the observed modifications of Bim_EL_. The level of an activated form of ERK, phospho Thr202/Tyr204, increased significantly 1 h after addition of garlic, declined slightly from 1–3 h, but was still higher than that of untreated cells after 3–24 h (Figure 6A). The kinase inhibitor PD98059, reduced the level of phospho-ERK to almost zero when added alone (Figure 6A). Phospho-ERK was also reduced when PD98059 was added in combination with DADS, but not to zero. Active forms of JNKs were also elevated after addition of garlic, but the highest levels occurred much later than was the case for ERK1/2. The amount of the activated forms of JNKs increased from 0 to 1 h was approximately equal from 1 to 7 h, and then increased further (16 and 24 h) (Figure 6B). A main band of JNK at 54 kDa and two minor bands at approximately 46 kDa were seen on Western blots. Others have also detected three JNK bands, probably representing different isoforms (Dreskin *et al*, 2001). The strongest pJNK band corresponded to the lowest JNK band. Phosphorylation of c-jun confirmed the activity of JNK (Figure 6B).

## DISCUSSION

A water-soluble extract of crushed garlic arrested MDA-MB-435 cancer cells in mitosis and caused apoptosis. The same effects have previously been shown for the garlic component SAMC on SW480 cells and NIH3T3 cells (Xiao *et al*, 2003). Destabilisation of the microtubules, resulting in incorrect spindle assembly, was probably the reason for the mitotic arrest after SAMC treatment. These effects resemble those of the cancer chemotherapy agents colcemid, nocodazole, and *Vinca* alkaloids. The molecular mechanisms resulting in apoptosis after treating cells with SAMC and other garlic compounds are, however, only partly understood and further studies of this process are important.

We have here characterised part of the molecular mechanism responsible for causing apoptosis after treating cells with a garlic extract. We focused on the proapoptotic BH3-only protein Bim, which previously has been shown to be important for apoptosis caused by another microtubule interfering agent, taxol (Bouillet *et al*, 1999; Chen and Zhou, 2004), and we show for the first time that a garlic extract had a clear effect on this protein. The level of a specific form of Bim_EL_ (band 3 on our Western blots) increased significantly 16 and 24 h after addition of garlic extract and the garlic compound DADS. Due to the position in the gel this form of Bim_EL_ could be identical to a form of Bim_EL_ (mBim_EL_) detected after treatment of cells with the microtubule-perturbation drug taxol and HIV-1 Tat protein (Chen and Zhou, 2004, Figure 1A). It was suggested that mBim_EL_ might be responsible for the observed strong dependence on Bim for both taxol- and Tat-induced apoptosis. We show that the form of Bim_EL_, which is increased as an effect of garlic extract and DADS, is phosphorylated. This specific phosphorylated form was relatively enriched in the mitochondrial fraction compared to the main form of Bim_EL_ in control cells (band2). The effect of garlic and DADS was therefore to increase the level of Bim_EL_ at the mitochondria, where it has its proapoptotic function. Another form of Bim_EL_ (band 1), which was increased after treatment with an inhibitor of ERK1/2 pathway, PD98059, was found only in the mitochondrial fraction. The increase in this form of Bim_EL_ could partly explain the enhanced apoptotic activity of the combination of garlic or DADS and PD98059 compared to garlic or DADS alone.

It is of interest that specific phosphorylations of Bim_L_ have been shown to influence the binding between this molecule and dynein light chain (LC-8/DLC1) (Lei and Davis, 2003). Phosphorylation of Thr-56 in Bim_L_ (corresponding to Thr-116 in Bim_EL_), caused by JNK, seems to release Bim from LC-8/DLC1, and this phosphorylation is probably negatively influenced by phosphorylation of Ser-58 (Ser-118 in Bim_EL_). ERK1/2 is theoretically a good candidate kinase for phosphorylation of the latter site (Scansite program, http://scansite.mit.edu/). We do not know the identity of the phosphorylated sites present in Bim_EL_ from MDA-MB-435 cells (band 2 and 3, Figure 3A), but Thr-116 and Ser-118, may be possible since these phosphorylations could be caused by JNK and ERK1/2, respectively, and because they probably influence the localisation of Bim.

If the modifications on Bim were of importance for apoptosis, downstream mitochondrial apoptotic effects should occur after these modifications, and the effects should be enhanced by the kinase inhibitor PD98059. An increase of cyt *c* in cytosol, which was enhanced by PD98059 and activation of caspase-9, related to the modifications of Bim in time, were indeed observed. The amount of a caspase-specific cleavage product of PARP was also enhanced by PD98059.

A temporary enhancement of active forms of ERK1/2 was observed during treatment of the cells with garlic. These kinases, however, had some activity also in untreated cells (Figure 6A). No change in the observed phosphorylation pattern of Bim_EL_ correlated with the temporary increased activation of ERK1/2. The activity of ERK1/2 in untreated cell was therefore probably enough for phosphorylation of the form of Bim_EL_ that was present in band 2. Inhibitors of ERK1/2 pathway has also been shown to enhance apoptosis caused by other microtubule-interfering agents already being used in cancer therapy, and the combinations are suggested to represent a new anticancer strategy, requiring lower drug dosages compared to drug monotherapy and may enhance tumor suppression *in vivo* (Zelivianski *et al*, 2003). Activation of JNK is reported to be necessary for apoptosis caused by different compounds of garlic in some cell lines (Filomeni *et al*, 2003; Xiao *et al*, 2003). Our results indicate that inhibition of JNK activity reduces the amount of Bim_EL_, especially a form of the protein that was enriched in the mitochondrial fraction. This effect may partly explain why apoptosis after treatment with compounds of garlic depends on JNK activity. Decreased level of Bim_EL_ after inhibition of JNK is in accordance with previous results, showing that JNK inhibitors inhibit the induction of Bim_EL_ and decrease the level of the protein (Putcha *et al*, 2003).

The garlic extract arrested MDA-MB-435 cells mainly in mitosis. It has previously been reported that the garlic compound SAMC arrested SW-480 and HT-29 cells both at G_2_ and mitosis (Shirin *et al*, 2001) and the reason for the mitotic arrest was probably disturbance of the mitotic spindle apparatus (Xiao *et al*, 2003). Furthermore it has been shown that DADS arrests HCT-15 cells (Knowles and Milner, 1998, 2000) and SH-SY5Y cells in G_2_/M (Filomeni *et al*, 2003). The arrest of the HCT-15 cells was probably a G_2_ arrest, since the activity of Cdc2 was decreased simultaneously. SH-SY5Y cells were at least partly arrested in mitosis since many cells with metaphasic chromosomes were detected. These results, taken together, indicate that the pattern of cell cycle arrest depends on the cell type and not on the specific garlic component used.

The garlic compound DADS, which has been used by other research groups to study apoptosis (Filomeni *et al*, 2003; Xiao *et al*, 2004), was also used to study details of the apoptotic process in MDA-MB-435 cells. It was found that this compound also caused modifications of Bim_EL_, similar to the effects of the garlic extract. Furthermore, DADS gave elevated levels of activated forms of ERK1/2 and a combination of an MEK/ERK inhibitor and DADS gave enhanced levels of an important apoptotic marker (85 kDa PARP-fragment) compared to DADS alone. In spite of these similarities, DADS is probably not the active apoptotic compound in the garlic extract since DADS is not soluble in water. It is known that approximately 70% of the thiosulphinates in garlic is allicin (Han *et al*, 1995). Allicin is however, rapidly metabolised and reacts quickly with a molecule of cysteine giving two molecules of SAMC (Lawson and Wang, 1993). DADS can also be transformed to SAMC. The effects on cells caused by the garlic extract are very similar to those of SAMC observed by others (Shirin *et al*, 2001; Xiao *et al*, 2003) suggesting that this component may be the main active compound in the extract used by us. Unfortunately we were not able to test the effect of SAMC directly, since this compound is not commercially available. The concentrations of the active components in the extracts can only be indirectly compared to the concentrations of pure components. However, the highest thiosulphinate concentration used in our study, 20 *μ*g ml^−1^, corresponds to approximately 100 *μ*M allicin, giving a maximum concentration of SAMC of 200 *μ*M, which is similar to that used by others (150–300 *μ*m) (Xiao *et al*, 2003).

Together our results reveal in more detail the mechanism for the initial phase of the apoptosis of MDA-MB-435 cells caused by garlic. The results fit well with previous observations in works using different components of garlic. It may be that disturbance of microtubules, caused by garlic components after some time promote translocation of the proapoptotic protein Bim_EL_ to the mitochondria, and JNK may be necessary for this event. Inhibition of the ERK1/2 pathway increases the level of a mitochondria-localised form of Bim_EL_ that may enhance its apoptotic effect.

## References

[bib1] Agarwal KC (1996) Therapeutic actions of garlic constituents. Med Res Rev 16: 111–124 8788216 10.1002/(SICI)1098-1128(199601)16:1<111::AID-MED4>3.0.CO;2-5

[bib2] Antlsperger DSM, Dirsch VM, Ferreira D, Su J-L, Kuo M-L, Vollmar AM (2003) Ajoene-induced cell death in human promyeloleukemic cells does not require JNK but is amplified by the inhibition of ERK. Oncogene 22: 582–589 12555071 10.1038/sj.onc.1206161

[bib3] Biswas SC, Greene LA (2002) Nerve growth factor (NGF) down-regulates the Bcl-2 homology 3 (BH3) domain-only protein Bim and suppresses its proapoptotic activity by phosphorylation. J Biol Chem 277: 49511–49516 12388545 10.1074/jbc.M208086200

[bib4] Bouillet P, Metcalf D, Huang DCS, Tarlinton DM, Kay TWH, Ksntgen F, Adams JM, Strasser A (1999) Proapoptotic Bcl-2 relative Bim required for certain apoptotic responses, leukocyte homeostasis, and to preclude autoimmunity. Science 286: 1735–1738 10576740 10.1126/science.286.5445.1735

[bib5] Bradford MM (1976) A rapid and sensitive method for the quantitation of microgram quantities of protein utilizing the principle of protein-dye binding. Anal Biochem 72: 248–254 942051 10.1016/0003-2697(76)90527-3

[bib6] Chen D, Zhou Q (2004) Caspase cleavage of Bim_EL_ triggers a positive feedback amplification of apoptotic signalling. Proc Natl Acad Sci USA 101: 1235–1240 14732682 10.1073/pnas.0308050100 PMC337036

[bib7] Cory S, Adams JM (2002) The Bcl-2 family: regulators of the cellular life-or-death switch. Nat Rev Cancer 2: 647–656 12209154 10.1038/nrc883

[bib8] Dirsch VM, Gerbes AL, Vollmar AM (1998) Ajoene, a compound of garlic, induces apoptosis in human promyeloleukemic cells, accompanied by generation of reactive oxygen species and activation of nuclear factor êB. Mol Pharmacol 53: 402–407 9495804 10.1124/mol.53.3.402

[bib9] Dreskin SC, Thomas GW, Dale SN, Heasley LE (2001) Isoforms of Jun kinase are differentially expressed and activated in human monocyte/macrophage (THP-1) cells. J Immunol 166: 5646–5653 11313405 10.4049/jimmunol.166.9.5646

[bib10] Ellison G, Klinowska T, Westwood RF, Docter E, French T, Fox JC (2002) Further evidence to support the melanocytic origin of MDA-MB-435. Mol Pathol 55: 294–299 12354931 10.1136/mp.55.5.294 PMC1187258

[bib11] Filomeni G, Aquilano K, Rotilio G, Ciriolo MR (2003) Reactive oxygen species-dependent c-jun NH2-terminal kinase/c-jun signalling cascade mediates neuroblastoma cell death induced by diallyl disulfide. Cancer Res 63: 5940–5949 14522920

[bib12] Gavrielli Y, Sherman Y, Ben-Sasson SA (1992) Identification of programmed cell death *in situ* via specific labelling of nuclear DNA fragmentation. J Cell Biol 119: 493–501 1400587 10.1083/jcb.119.3.493 PMC2289665

[bib13] Han J, Lawson L, Han G, Han P (1995) A spectrophotometric method for quantitative determination of allicin and total garlic thiosulfinates. Anal Biochem 225: 157–160 7778769 10.1006/abio.1995.1124

[bib14] Huang DCS, Strasser A (2000) BH3-only proteins – essential initiators of apoptotic cell death. Cell 103: 839–842 11136969 10.1016/s0092-8674(00)00187-2

[bib15] Knowles LM, Milner JA (1998) Depressed p34^cdc2^ kinase activity and G_2_/M phase arrest induced by diallyl disulfide in HCT-15 cells. Nutr Cancer 30: 169–174 9631486 10.1080/01635589809514659

[bib16] Knowles LM, Milner JA (2000) Diallyl disulfide inhibits p34^cdc2^ kinase activity through changes in complex formation and phosphorylation. Carcinogenesis 21: 1129–1134 10837000

[bib17] Larsen JK, Munch-Petersen B, Christiansen J, Jørgensen K (1986) Flow cytometric discrimination of mitotic cells: Resolution of M, as well as G_1_, S and G_2_ phase nuclei with mithramycin, propidium iodide, ethidium bromide after fixation with formaldehyde. Cytometry 7: 54–63 2419056 10.1002/cyto.990070108

[bib18] Lawson LD, Wang ZJ (1993) Pre-hepatic fate of the organosulfur compounds derived from garlic (*Alllium sativum*). Planta Med 59(Suppl): A688–A689

[bib19] Lei K, Davis RJ (2003) JNK phosphorylation of Bim-related members of the Bcl-2 family induces Bax-dependent apoptosis. Proc Natl Acad Sci USA 100: 2432–2437 12591950 10.1073/pnas.0438011100 PMC151358

[bib20] Ley R, Balmanno K, Hadfield K, Weston C, Cook SJ (2003) Activation of the ERK1/2 signaling pathway promotes phosphorylation and proteasome-dependent degradation of the BH3-only protein, Bim. J Biol Chem 278: 18811–18816 12646560 10.1074/jbc.M301010200

[bib21] Luciano F, Jacquel A, Colosetti P, Herrant M, Cagnol S, Pages G, Auberger P (2003) Phosphorylation of Bim_EL_ by ERK1/2 on serine 69 promotes its degradation via the proteasome pathway and regulates its proapoptotic function. Oncogene 22: 6785–6793 14555991 10.1038/sj.onc.1206792

[bib22] Marani M, Hancock D, Lopes R, Tenev T, Downward J, Lemoine NR (2004) Role of Bim in the survival pathway induced by Raf in epithelial cells. Oncogene 23: 2431–2441 14676826 10.1038/sj.onc.1207364

[bib23] Marani M, Tenev T, Hancock D, Downward J, Lemoine NR (2002) Identification of novel isoforms of the BH3 domain protein Bim which directly activate bax to trigger apoptosis. Mol Cell Biol 22: 3577–3589 11997495 10.1128/MCB.22.11.3577-3589.2002 PMC133811

[bib24] Nakagawa H, Tsuta K, Kiucki K, Senzaki H, Tanaka K, Tsubura A (2001) Growth inhibitory effects of diallyl disulfide on human breast cancer cell lines. Carcinogenesis 22: 891–897 11375895 10.1093/carcin/22.6.891

[bib25] O’Connor L, Strasser A, O’Reilly LA, Hausmann G, Adams JM, Cory S, Huang DCS (1998) Bim-a novel member of the Bcl-2 family that promotes apoptosis. EMBO J 17: 384–395 9430630 10.1093/emboj/17.2.384 PMC1170389

[bib26] O’Connor PM, Jackman J, Bae I, Myers TG, Fan S, Mutoh M, Scudier DA, Monks A, Sausville EA, Weinstein JN, Friend S, Fornace Jr AJ, Kohn KW (1997) Characterization of the p53 tumor suppressor pathway in cell lines of the National Cancer Institute anticancer drug screen and correlations with the growth-inhibitory potency of 123 anticancer agents. Cancer Res 57: 4285–4300 9331090

[bib27] O’Reilly LA, Cullen L, Visvader J, Lindeman GJ, Print C, Bath ML, Huang DCS, Strasser A (2000) The proapoptotic BH3-only protein Bim is expressed in hematopoietic, epithelial, neuronal, and germ cells. Am J Pathol 157: 449–461 10934149 10.1016/S0002-9440(10)64557-9 PMC1850143

[bib28] Putcha GV, Le S, Frank S, Besirli CG, Clark K, Chu B, Alix S, Youle RJ, LaMarche A, Maroney AC, Johnson Jr EM (2003) JNK-mediated Bim phosphorylation potentiates BAX-dependent apoptosis. Neuron 38: 899–914 12818176 10.1016/s0896-6273(03)00355-6

[bib29] Puthalakath H, Huang DCS, O’Reilly LA, King SM, Strasser A (1999) The proapoptotic activity of the Bcl-2 family member Bim is regulated by interaction with the dynein motor complex. Mol Cell 3: 287–296 10198631 10.1016/s1097-2765(00)80456-6

[bib30] Seward RJ, von Haller PD, Aebersold R, Huber BT (2003) Phosphorylation of the pro-apoptotic protein Bim in lymphocytes is associated with protection from apoptosis. Mol Immunol 39: 983–993 12749905 10.1016/s0161-5890(03)00047-6

[bib31] Shirin H, Pinto JT, Kawabata Y, Soh J-W, Delohery T, Moss SF, Murty V, Rivlin RS, Holt PR, Weinstein IB (2001) Antiproliferative effects of S-allylmercaptocysteine on colon cancer cells when tested alone or in combination with sulindac sulphide. Cancer Res 61: 725–731 11212275

[bib32] Sivam GP, Lampe JW, Ulness B, Swanzy SR, Potter JD (1997) *Helicobacter pylori* – *in vitro* susceptibility to garlic (*Allium sativum*) extract. Nutr Cancer 27: 118–121 9121937 10.1080/01635589709514512

[bib33] Stokke T, Solberg K, DeAngelis P, Steen HB (1998) Propidium iodide quenches the fluorescence of TdT incorporated FITC-labeled dUTP in apoptotic cells. Cytometry 33: 428–434 9845437

[bib34] Wang P, Gilmore AP, Streuli CH (2004) Bim is an apoptosis sensor that responds to loss of survival signals delivered by Epidermal Growth Factor but not those provided by integrins. J Biol Chem 279: 41280–41285 15292207 10.1074/jbc.C400248200

[bib35] Xiao D, Choi S, Johnson DE, Vogel VG, Johnson CS, Trump DL, Lee YL, Singh SV (2004) Diallyl trisulfide-induced apoptosis in human prostate cancer cells involves c-Jun N-terminal kinase and extracellular-signal regulated kinase-mediated phosphorylation of Bcl-2. Oncogene 23: 5594–5606 15184882 10.1038/sj.onc.1207747

[bib36] Xiao D, Pinto JT, Soh J-W, Deguchi A, Gundersen GG, Palazzo AF, Yoon J-T, Shirin H, Weinstein BI (2003) Induction of apoptosis by the garlic-derived compound s-Allylmercaptocysteine (SAMC) is associated with microtubule depolymerization and c-jun NH2-terminal kinase 1 activation. Cancer Res 63: 6825–6837 14583480

[bib37] Zelivianski S, Spellman M, Kellerman M, Kakitelashvilli V, Zhou XW, Lugo E, Lee MS, Taylor R, Davis TL, Hauke R, Lin MF (2003) ERK inhibitor PD98059 enhances docetaxel-induced apoptosis of androgen-independent human prostate cancer cells. Int J Cancer 107: 478–485 14506750 10.1002/ijc.11413

